# Detection of evolutionary conserved and accelerated genomic regions related to adaptation to thermal niches in *Anolis* lizards

**DOI:** 10.1002/ece3.11117

**Published:** 2024-03-07

**Authors:** Fuku Sakamoto, Shunsuke Kanamori, Luis M. Díaz, Antonio Cádiz, Yuu Ishii, Katsushi Yamaguchi, Shuji Shigenobu, Takuro Nakayama, Takashi Makino, Masakado Kawata

**Affiliations:** ^1^ Graduate School of Life Sciences Tohoku University Sendai Japan; ^2^ National Museum of Natural History of Cuba Havana Cuba; ^3^ Faculty of Biology University of Havana Havana Cuba; ^4^ Trans‐Omics Facility National Institute for Basic Biology Okazaki Japan; ^5^ Department of Basic Biology, School of Life Science The Graduate University for Advanced Studies, SOKENDAI Okazaki Japan; ^6^ Division of Life Sciences, Center for Computational Sciences University of Tsukuba Tsukuba Japan; ^7^ Present address: Department of Biology University of Miami Coral Gables Florida USA

**Keywords:** accelerated evolution, *Anolis*, comparative genomics, reptiles, thermal adaptation

## Abstract

Understanding the genetic basis for adapting to thermal environments is important due to serious effects of global warming on ectothermic species. Various genes associated with thermal adaptation in lizards have been identified mainly focusing on changes in gene expression or the detection of positively selected genes using coding regions. Only a few comprehensive genome‐wide analyses have included noncoding regions. This study aimed to identify evolutionarily conserved and accelerated genomic regions using whole genomes of eight *Anolis* lizard species that have repeatedly adapted to similar thermal environments in multiple lineages. Evolutionarily conserved genomic regions were extracted as regions with overall sequence conservation (regions with fewer base substitutions) across all lineages compared with the neutral model. Genomic regions that underwent accelerated evolution in the lineage of interest were identified as those with more base substitutions in the target branch than in the entire background branch. Conserved elements across all branches were relatively abundant in “intergenic” genomic regions among noncoding regions. Accelerated regions (ARs) of each lineage contained a significantly greater proportion of noncoding RNA genes than the entire multiple alignment. Common genes containing ARs within 5 kb of their vicinity in lineages with similar thermal habitats were identified. Many genes associated with circadian rhythms and behavior were found in hot‐open and cool‐shaded habitat lineages. These genes might play a role in contributing to thermal adaptation and assist future studies examining the function of genes involved in thermal adaptation via genome editing.

## Graphical abstract

Whole genomes were used from eight *Anolis* lizard species that repeatedly adapted to similar thermal habitats in multiple lineages and we identified evolutionarily conserved or accelerated genomic regions.

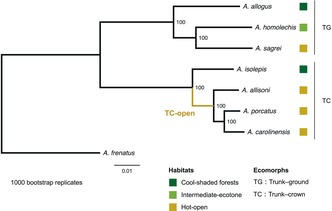

## 1 INTRODUCTION

Climate change, such as global warming, has significantly impacted many organisms (Parmesan, 2006; Root et al., 2003; Walther et al., 2002). Ectotherms, the organisms depending on external heat sources for regulating body temperature, are highly sensitive to climate change (Burraco et al., 2020). Sinervo et al. (2010) assessed the extinction risks of lizard species associated with global warming and predicted their 20% extinction by 2080. Thermoregulatory function varies among lizard lineages (Grigg & Buckley, 2013) and may constrain the response to climate change. Several studies (Araújo et al., 2013; Muñoz et al., 2014) suggested that more evolution constraints may occur with tolerance to higher temperatures than with tolerance to lower temperatures. Many tropical ectothermic species are already living close to their maximum temperature limits and may be vulnerable to even slight temperature rise (Deutsch et al., 2008; Muñoz et al., 2014). Several ectothermic species show rapidly decreasing fitness above the maximum fitness temperature (Buckley et al., 2022; Martin & Huey, 2008), and this change is usually more abrupt at lower temperatures. Considering the asymmetry of the temperature adaptation curve, a slightest increase in habitat temperature can threaten the species survival. Even if the average habitat temperature is below the maximum fitness temperature, the temperature variance experienced by individuals in their actual habitat may decrease fitness (Martin & Huey, 2008). Climate change is expected to have complex effects on the populations of ectothermic animals worldwide, and forest‐dwelling tropical lizards are more vulnerable to global warming than species living in open areas (Huey et al., 2009). Identifying the adaptive evolutionary processes for environments from genomic information could be an important approach for predicting the potential viability of these populations (Hoffmann et al., 2021). Rapid climate change exerts selective pressures on populations, and evolutionary adaptation can play an crucial role in responding to this change (Hoffmann & Sgrò, 2011). Therefore, identifying the past adaptive evolution to thermal environments by genomic comparison of ectothermic species will provide useful information for evaluating the possible evolutionary adaptations to future environmental changes.

Populations or species with different habitat temperatures can exhibit different thermal traits (Brusch et al., 2016; Gutiérrez‐Pesquera et al., 2016; Li et al., 2017), and differences in thermal traits at different temperatures may result from genetic factors and phenotypic plasticity (Domínguez‐Guerrero et al., 2021; Drummond et al., 2020; Ryan & Gunderson, 2021). CT_max_ and CT_min_ indicate the upper and lower temperature limits of the activity of a species, respectively, and demonstrate higher heritability than the thermal optimum and performance breadth (Logan & Cox, 2020). Studies comparing urban and forest populations of *Anolis cristatellus* demonstrated repeated selection of CT_max_‐related genes, and genetic divergence in the genes related to adaptive and nonadaptive plasticity between urban and forest populations (Campbell‐Staton et al., 2021). The evolutionary potential and thermo‐adaptability mode in ectothermic organisms remains unclear; however, gradual understanding of association between thermal physiology and genomics is being gained by combining high‐throughput sequencing methods and phenotypic analyses.

*Anolis* lizards in the Caribbean islands have diversified by adapting to various structural microhabitats, resulting in the evolution of distinct set of species known as ectomorphs. Ecomorphs share a similar structural habitat, morphology, ecology, and behavior but do not essentially belong to only one lineage (Losos, 2009; Williams, 1972). In addition, several *Anolis* lizards of same ecomorph have diversified into thermally distinct habitats (Cádiz et al., 2013; Hertz et al., 2013; Huey & Webster, 1976; Ruibal, 1961). In Cuba, several *Anolis* species of the same ecomorph dwell in the same forest throughout the island, although with different thermal habitats (Cádiz et al., 2013; Ruibal, 1961). Different thermal environments occur within the same location in Cuba such as cool‐shaded forests, intermediate‐ecotone habitats, and hot‐open habitats. In cool‐shaded forests, the ambient temperature remains relatively cool as direct sunlight barely reaches the forest floor while in hot‐open habitats or human‐made open areas, the temperature is higher due to prolonged exposure to direct sunlight. In intermediate‐ecotone habitats, the ambient temperature along the forest edges is intermediate compared with that in deep forest and open areas. Different thermal environments and sunlight basking opportunities in these habitats have resulted in species‐specific average field body temperatures in these *Anolis* species (Ruibal, 1961; Schettino et al., 2010) indicating divergence in their preferences and physiological performance in different thermal environments. Among trunk–ground ecomorph species living on tree trunks and ground, *Anolis sagrei* inhabits a hot‐open habitat, *A. homolechis* prefers an intermediate‐ecotone habitat at the forest edge, and *A. allogus* resides in cool‐shaded forests. Among the trunk–crown ecomorph species residing on tree trunks and branches, *A. porcatus* and *A. allisoni* inhabit hot‐open habitats, whereas *A. isolepis* lives in cool‐shaded forests (Ruibal, 1961; Schettino, 1999).

*Anolis sagrei*, *A. allisoni*, and *A. porcatus* have become invasive species in non‐native habitats (Kolbe et al., 2004, 2007; Krysko et al., 2015). *Anolis carolinensis*, the only *Anolis* species native to North America and closely related to *A. porcatus* (Glor et al., 2005), has invaded Hawaii, the Ogasawara Islands, and Okinawa in Japan (Suzuki‐Ohno et al., 2017; Tamate et al., 2017). These species are found in hot‐open areas, and their ability to adapt to novel environments is associated with these habitats.

In addition to the above examples, many studies have assessed the thermal niche of *Anolis* lizards. Thermal traits and energy consumption vary among habitat thermal environments (Gunderson et al., 2018; Rogowitz, 2003). Muñoz et al. (2014) compared the thermal traits of *Anolis* species at different habitat elevations and temperatures and showed that CT_min_ evolution was significantly faster than CT_max_ evolution. The natural environment of a habitat has been reported to influence the ecomorph composition of *Anolis* lizards (Wollenberg et al., 2013). Therefore, focusing on species that inhabit similar thermal niches among various ecomorphs would be an effective way to explore their adaptation mechanisms to the habitat temperature of the environment beyond the framework of ecomorph composition.

The genetic basis for thermal adaptation in *Anolis* lizards has been described in several studies (Akashi et al., 2016, 2018; Campbell‐Staton et al., 2017, 2018, 2020; Kanamori et al., 2021; Rodríguez et al., 2017). Akashi et al. (2016) identified differentially expressed genes (DEGs) between various thermal conditions in three Cuban *Anolis* species (*A. allogus*, *A. homolechis*, and *A. sagrei*) and found that genes associated with circadian regulation were one of the commonly detected DEGs among the three species. Of these, *NR1D1*, which encodes the nuclear receptor REV‐ERB‐α and is involved in the circadian regulation of metabolic activity, exhibited opposing expression patterns in *A. allogus* (living in a cool‐shaded habitat) and *A. sagrei* (living in a hot‐open habitat). Akashi et al. (2018) also found significant difference between the experimental voluntary maximum, defined as the temperature at which organisms escape from a heat source, in *A. allogus* and *A. sagrei*. The activation temperature and amino acid sequence of the temperature‐sensitive channel TRPA1 differed between the two species. Kanamori et al. (2021) identified differences in each lineage in the genes with positive natural selection in protein‐coding regions in two hot‐open habitat lineages; however, those related to DNA repair, oxidative stress, and cardiac function were present in both lineages. However, few adaptive evolution studies have encompassed the entire genome, including introns and intergenic regions and this study focuses only on coding regions.

Recently, whole genomes have been established for several *Anolis* species (Alföldi et al., 2011; Kanamori et al., 2022; Tollis et al., 2018), and data for comparative analyses of noncoding regions have become available. Noncoding regions of the genome contain various functional elements, such as cis‐regulatory regions (Wittkopp & Kalay, 2011; Wray, 2007). Identifying functional sites in these regions is also important for understanding the genetic basis of thermal adaptation and detecting evolutionarily conserved or accelerated genomic regions may help identify regions that evolve under natural selection. Regions with highly conserved sequences across lineages are expected to be under strong selective constraints because of their functional importance (Lindblad‐Toh et al., 2011; Seki et al., 2017). Comparative genomic analysis using up to 240 mammalian genomes identified ultraconserved bases (Christmas et al., 2023). Of significantly constrained single bases, 80% were in noncoding regions and 50% were in previously unannotated regions. Evolutionarily constrained bases were more likely to overlap with human trait‐associated mutations than other annotations (Sullivan et al., 2023). Searching evolutionarily conserved regions over lineages can provide candidates for previously unknown functional elements in the genome. Evolutionary accelerated genomic regions have significantly higher than expected base substitution rates and may reflect positive selection or relaxation of negative selection (Hubisz & Pollard, 2014). Therefore, information on accelerated evolution can be used to identify genomic regions that undergo natural selection and are involved in lineage‐specific phenotypes. Accelerated genomic regions have been shown to contribute to various phenotypes (Kvon et al., 2016; Levchenko et al., 2018) or have indicated the functional noncoding regions (Capra et al., 2013; Ferris et al., 2018; Holloway et al., 2016).

Herein, we identified candidate genes that may be relevant to thermal microhabitat adaptation from the genome sequences of *Anolis* lizards. These genes could be candidates for subsequent functional analysis, for example, via gene editing. For this purpose, evolutionarily conserved or accelerated genomic regions were identified using the genomes of six Cuban *Anolis* species (*A. sagrei*, *A. homolechis*, *A. allogus*, *A. porcatus*, *A. allisoni*, and *A. isolepis*) and their closely related species (*A. carolinensis*) that live in different thermal environments. Common genes containing ARs within or near themselves in lineages with similar habitats (hot‐open habitats or cool‐shaded forests) were also identified.

## 2 MATERIALS AND METHODS

### 2.1 Genomic data

Genomic data were used from six Cuban *Anolis* species (Kanamori et al., 2022); Illumina short‐read sequence data are available at the DDBJ Short Read Archive database (https://www.ddbj.nig.ac.jp/dra/index.html) with accession number DRA013941. Sets of the 10× barcode fast files, soft‐masked genome assemblies, gene models, and predicted coding nucleotide and peptide sequences are available at Figshare (https://figshare.com/projects/Reconstruction_of_draft_genomes_for_six_Cuban_Anolis_lizards/137421). The six genomes included three trunk–ground species (*A. sagrei*, *A. homolechis*, and *A. allogus*) and three trunk–crown species (*A. porcatus*, *A. allisoni*, and *A. isolepis*). *A. allogus* and *A. isolepis* reside in cool‐shaded forests, *A. sagrei*, *A. porcatus*, and *A. allisoni* live in hot‐open habitats, and *A. homolechis* is found in intermediate‐ecotone habitats (See Table S1 for details on the habitat and temperature characteristics of the Cuban *Anolis* species used in this study). The 13 established chromosomes of *A. carolinensis*, which is closely related to *A. porcatus* (Glor et al., 2005), were added to the dataset (AnoCar2.0v2, GenBank assembly accession: GCA_000090745.2). The genome of *A. frenatus* (Tollis et al., 2018) was used as an outgroup species for phylogenetic analyses. Whole genome sequences of the eight species were used for genome alignment. The coding DNA sequences (CDS) and nucleotide and amino acid sequences were used to visualize codon usage and establish a neutral evolutionary model as a basis for calculating evolutionary conservation and acceleration in genomic regions. For *A. frenatus*, CDS nucleotide sequence data were generated from the genome sequence and via genome annotation using AGAT v0.6.0 (https://www.doi.org/10.5281/zenodo.3552717).

### 2.2 Whole genome alignment

Multiple alignments for each genomic region were constructed for comparing evolutionarily homologous genomic regions throughout the *Anolis* lizard genomes. Pairwise alignments of the genome for each species with the *A. carolinensis* genome were generated using LastZ v1.04.03 (Harris, 2007). Initial pairwise alignments were extended by joining alignments of neighboring regions according to the “chaining and netting” (Kent et al., 2003). The quality of the alignment was improved by filtering the alignments using our own scripts that executed the following processes: (1) deletion of ambiguous bases denoted by N as in the following cases: Ns appearing at either edge of an alignment and N‐repeat located in the middle of an aligned sequence and occupying >50% of the length of the sequence; (2) selection of a multiple aligned region. Sometimes, multiple target sequences were aligned with an identical sequence of the *A. carolinensis* genome and syntenic relationships are ambiguous in such cases. Sequences appearing more plausible as homologous regions to the reference were selected to manufacture a single unified alignment for each area. A longer alignment was preferentially adopted. If multiple target sequences overlapped the same reference region, the overlap of the shorter alignment was removed. Adopting the approach that favored more broadly consistent alignments that maintain context, enhanced the selection of syntenic regions. A single alignment was created for each area by repeating this procedure.

Pairwise alignments were merged into multiple alignments using MultiZ. v11.2 (Blanchette et al., 2004). A phylogenetic tree estimated by Cádiz et al. (2018) was provided as a guide tree topology for multiple alignments using MultiZ. Multiple alignments were filtered using our own script to only select alignments that included sequences from more than seven of eight species.

### 2.3 Estimation of the neutral evolutionary model

**FIGURE 1:**
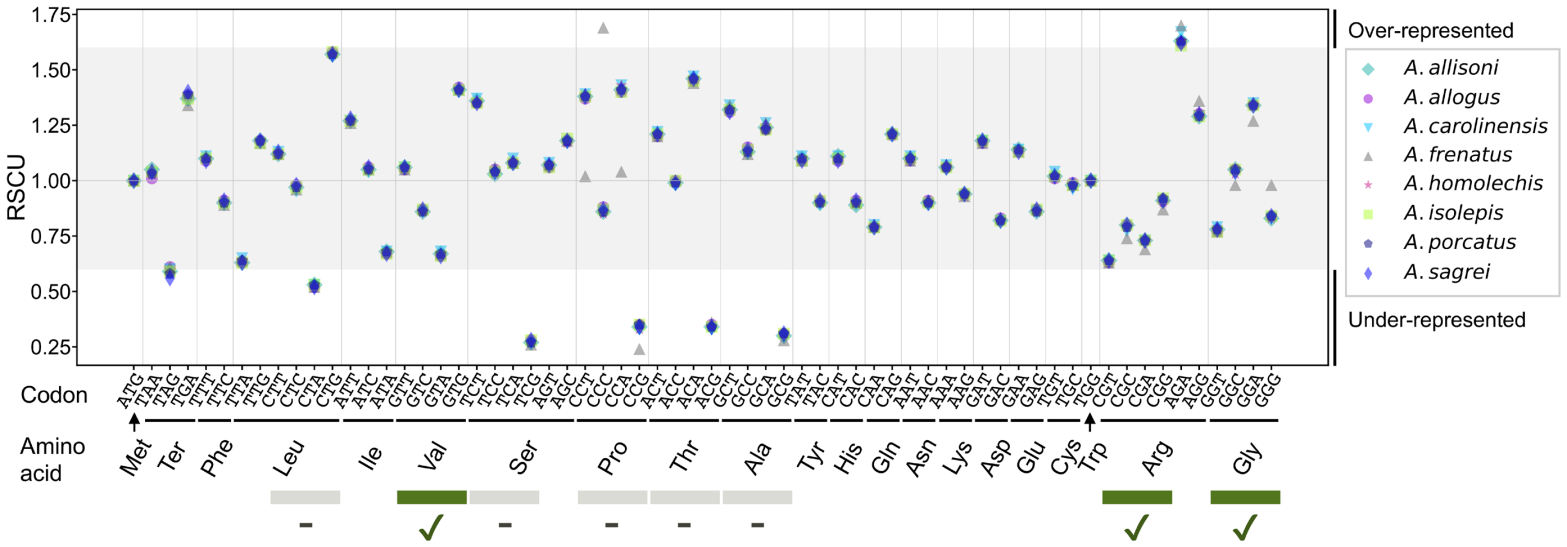
Relative synonymous codon usage (RSCU). Plots on the white background indicate underrepresented (<0.6) or overrepresented (>1.6) codons. The bottom square covers the 4D sites, “✓” indicates codons used for the neutral model estimation, and “‐” indicates codons excluded from the estimation of the neutral model. 4D site, Fourfold degenerate site.

The neutral evolutionary model of *Anolis* lizards was used as a reference model (null model) for detecting genomic regions that are conserved or underwent accelerated evolution. Fourfold degenerate sites (4D sites), a set of third sites of a codon where every possible mutation is synonymous, have been widely used for estimating the neutral evolutionary model assuming neutral evolution (Pheasant & Mattick, 2007). However, recent studies suggest effect of a synonymous codon change on translation, protein conformation, or other factors (Hanson & Coller, 2018) indicating that nucleotide changes at 4D sites are not necessarily neutral. Therefore, although a neutral evolutionary model based on 4D sites was estimated, sites with extreme usage bias (Figure 1) were excluded. The usage bias of 4D sites was detected in following steps. First, single‐copy orthologs were detected by OrthoFinder v2.5.4 in the eight *Anolis* species using the amino acid sequence (Emms & Kelly, 2019). Relative synonymous codon usage (RSCU) in the codon set encoding the same amino acid was calculated for 4316 sets of single‐copy genes. According to previous reports (Feng et al., 2020; Wong et al., 2010), codons with an RSCU <0.6 or >1.6 were considered “biased” in usage frequency. Consequently, among the codon sets containing 4D sites, bias in usage was found for CTA encoding Leu, TCG encoding Ser, CCG encoding Pro, ACG encoding Thr, and GCG encoding Ala in all species (RSCU <0.6). Therefore, these five codon sets were excluded from subsequent analyses, and the three sets of GT*, CG*, and GG* (* is either A, T, G, or C) encoding Val, Arg, and Gly, respectively, were used to estimate the neutral evolutionary model as unbiased 4D sites. The CDS of single‐copy genes using PRANK v170703 (Löytynoja, 2014) was aligned and the unbiased 4D sites were extracted from the alignment using our own script. The extracted 282,352 4D sites underwent maximum likelihood phylogenetic tree estimation using Randomized Axelerated Maximum Likelihood (RAxML) v0.9.0 (Stamatakis, 2014) as a single dataset. The base substitution model used for the phylogenetic estimation was GTR + G4, and bootstrap sampling was performed 1000 times. Finally, a neutral evolutionary model of *Anolis* lizards was established using phyloFit in the PHAST v1.4 software package (Hubisz et al., 2011) with the unbiased 4D sites and the topology obtained in the maximum likelihood phylogenetic tree estimation.

### 2.4 Detection of genomic regions that underwent conserved or accelerated evolution

Multiple alignments of partial genome sequences produced using MultiZ (see Section 2.2) were divided and classified into five categories based on gene annotation of the *A. carolinensis* genome (AnoCar2.0). The four annotation categories were as follows: (1) CDS, (2) 5′‐untranslated region (5′‐UTR), (3) 3′‐untranslated region (3′‐UTR), and (4) introns. All other alignments that did not fit into above categories were classified as an intergenic sequence in category 5. If a genomic region fitted into more than one category, that is, the corresponding location on the *A. carolinensis* is labeled as multiple annotation categories, it was assigned to the divided alignment in a single category in the following order: CDS, 5′‐UTR, 3′‐UTR, intron, and intergenic (Siepel et al., 2005). This is referred in following cases: same region is annotated multiple times within one gene or is annotated with a different gene. The former case occurs when splicing variants or multiple starting positions of a gene are registered, and consequently, the alignment was divided at the annotation boundary and each region was assigned one high‐priority annotation. Alternatively, the annotation was also adopted in the order of priority in the latter case; however, if a particular region encodes multiple genes and is annotated as the same category in both genes such as, if a region overlapped the CDS of two genes, a common annotation was assigned for a region and both gene IDs were considered. In addition, the statistical power for acceleration/conservation detection decreases for very short region lengths and very long region lengths mask the smaller acceleration/conservation signals (Pollard et al., 2010; Siepel et al., 2005). Therefore, the region lengths were adjusted to 10–99 bases in this analysis. The average length of ARs found in humans is approximately 260 bp (Levchenko et al., 2018). The average length of conserved regions found in vertebrates, insects, and yeasts is approximately 100–120 bp (Siepel et al., 2005); therefore, the region length was set to avoid obscuring the conserved regions/ARs of several 100 bp. The window size for detecting acceleration and conservation was adjusted to 10–99 bases based on the studies selecting a window of approximately 50 bp (Ferris et al., 2018; Ferris & Gregg, 2019). The alignment was split every 60 bases from a tip using an in‐house script for alignments of ≥100 bases, but no fragments of <20 bp were produced at the last split. Alignments with lengths of 10–99 bases were not processed in this step and were used for the subsequent process “as is,” whereas alignments <10 bases in length were removed because of the possibility of being of low quality. These annotated windows with 10–99 bases were used for the analyses. After splitting the multiple alignments by length, the number of windows increased from 920,406 to 9,655,872, and the median window length changed from 315 to 60.

The estimated neutral model was used as the null model and *p*‐values for alternative hypothesis acceptance (conservation or acceleration) were calculated per window of the multiple alignments by using phyloP (Pollard et al., 2010) in PHAST v1.4 package. For phyloP, the input sequences of each genomic window in a multiple alignment were compared with a neutral model for testing the presence of a conservation or acceleration trend (i.e., the number of base substitutions is relatively small or large) across all sample species or at a branch of interest. The conservation‐acceleration (CONACC) scores for each genomic region were calculated using phyloP for confirming the overall conservation/acceleration trend for each annotation category of each lineage. The CONACC score is obtained by converting the acceleration *p*‐values into negative values and the conservation *p*‐values into positive values, which illustrates conservation and acceleration as consecutive values. This allows determination of acceleration or conservation side of each region compared with a neutral model.

The windows of multiple alignments with two evolutionary trends were detected: (1) regions with a high average degree of sequence conservation across all branches in the phylogenetic tree, hereafter referred as all‐branch conserved elements (all‐branch CEs); and (2) regions predicted to be rapidly evolving in the branch of interest compared with all other lineages in the background, hereafter referred as accelerated regions (ARs). All‐branch CEs are considered as potentially important genomic regions throughout the lizard species analyzed in this study. However, ARs may include genomic regions contributing in the evolution of individual lineages, including that of their respective ecomorphs and/or adaptation to each thermal habitat. ARs were essentially detected at the branches directly leading to each species; however, because *A. carolinensis*, *A. porcatus*, and *A. allisoni* share the same thermal habitat (hot‐open), ecomorph (trunk–crown), and comprised a single clade (trunk–crown open: TC‐Open), ARs were also detected at the branch leading to the latest common ancestor of these three species along with at each branch of these species. Accounting for multiple comparisons, the calculated *p*‐values for conservation over the entire lineage and those for acceleration in each target branch were false discovery rate (FDR)‐corrected using the Benjamini–Hochberg technique with the “multipletests” function included in the Python module “statsmodels” (Seabold & Perktold, 2010). Regions with an FDR <0.05 were detected as all‐branch CEs and ARs.

### 2.5 Analysis of genes associated with evolutionary conserved and accelerated regions

Gene annotation data from the *A. carolinensis* genome (AnoCar2.0) was used to gain insight into detected accelerated/conserved genomic regions by surveying genes that may have been affected by accelerated and conserved evolution. For ARs with the intron or intergenic category, those overlapping with all‐branch CEs were used with the assumption that all‐branch CEs represent putative functional elements. All‐branch CEs could be functionally essential, and ARs within all‐branch CEs may indicate accelerated evolution in functional regions. When a detected sequence was annotated either as a CDS, 5′‐/3′‐UTR, or intron, information for the gene associated with that annotation was retrieved. For assigned “intergenic” regions, the “intergenic” region within 5 kb upstream or downstream of the gene was extracted as a region that may affect the expression regulation of the gene, considering ChIP‐seq data, DNA methylation levels, open chromatin information, and histone marks in avian‐conserved regions near genes (Deng et al., 2009; McLean et al., 2010; Seki et al., 2017; Wang et al., 2018). Regions within 5 kb upstream or downstream of the gene were collected using the “flank” command of bedtools v2.30.0 (Quinlan & Hall, 2010), and the conserved regions or ARs overlapping with the region near the gene were extracted using the “intersect” command of bedtools v2.30.0 (Quinlan & Hall, 2010). Any incomplete data for the associated genes, including names or functional annotation of the product, in the *A. carolinensis* genome annotation, was complemented by using the corresponding information of orthologs in other organisms (i.e., chicken, human, or mouse) in the Ensembl release 106.

To identify important genomic regions for adaptive evolution in thermal environments, we extracted common genes that included ARs among lineages with similar thermal microhabitats. The lineages included two of hot‐open habitats (TC‐open and *A. sagrei*) and two of cool‐shaded habitats (*A. isolepis* and *A. allogus*). We collected gene names not only when the same window of multiple alignment was accelerated in the two lineages but also when ARs existed across multiple annotation categories within the same gene.

### 2.6 Enrichment analysis of putative functional elements in evolutionary conserved or accelerated regions

Based on *A. carolinensis* genome annotation, all‐branch CEs or ARs with >80% of the sequence overlapping with noncoding RNA (ncRNA) genes, including those for small nuclear, long noncoding (lncRNA), micro, ribosomal, small nucleolar, and small conditional RNA were extracted using the “intersect” command of bedtools v2.30.0 (Quinlan & Hall, 2010). Fisher's exact probability test was applied to compare the number of windows containing ncRNA genes between the entire multiple alignment and all‐branch CEs or ARs. Gene Ontology (GO) enrichment analysis was performed for genes with ARs using PANTHER (http://www.pantherdb.org/; Thomas et al., 2003). The analysis type was “PANTHER Overrepresentation Test” with Fisher's exact test, and each *p*‐value obtained by GO enrichment analysis was FDR corrected to detect GO terms with an FDR <0.05.

## 3 RESULTS

### 3.1 Conservation and acceleration scores

**FIGURE 2:**
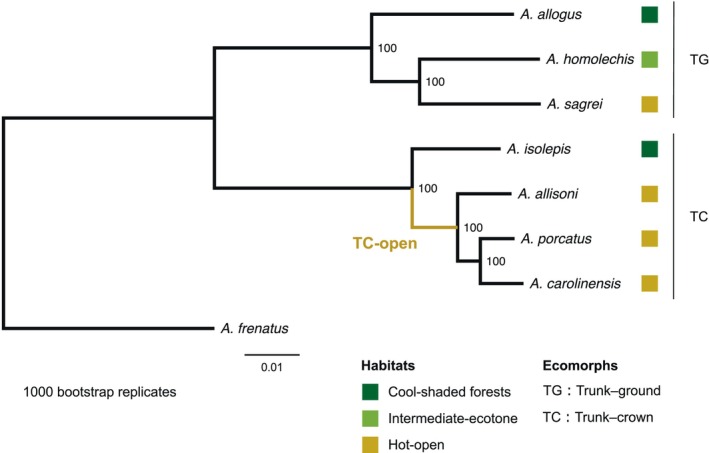
Neutral phylogenetic tree estimated from corrected 4D sites. The dark green, green, and yellow squares indicate cool‐shaded, intermediate‐ecotone, and hot‐open habitats, respectively. The colored branch indicates the branch leading to the common ancestor of the hot‐open species in the trunk–crown clade (TC‐open). The bar to the right indicates the ecomorphic clade. Numbers at the nodes indicate bootstrap values. 4D site, Fourfold degenerate site; TC, trunk–crown clade; TG, trunk–ground clade.

**FIGURE 3:**
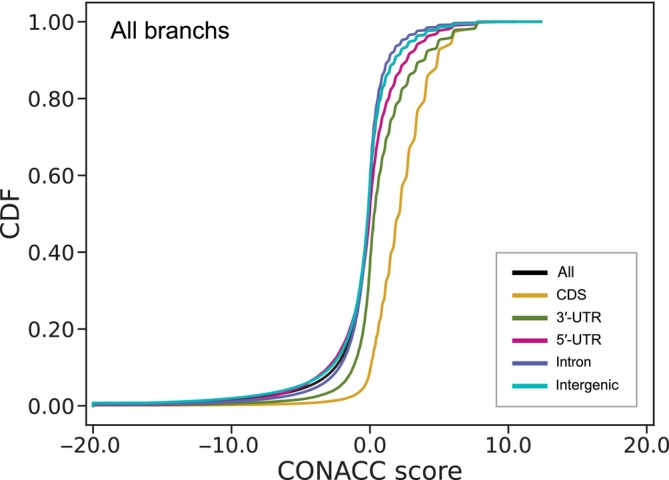
Cumulative distribution frequency (CDF) of the CONACC scores (conservation‐acceleration scores) of all branches. Positive larger CONACC scores represent more conserved and negative larger CONACC scores represent more acceleration. Color legends: all regions = black, CDS = yellow, 3′‐UTR = green, 5′‐UTR = magenta, intron = violet, and intergenic = cyan. 3′‐UTR, 3′‐untranslated region; 5′‐UTR, 5′‐untranslated region; CDS, protein coding sequence.

We identified regions containing highly conserved nucleotide sequences across all evaluated *Anolis* species and fast‐evolving regions with high nucleotide substitution rates in the branches of each Cuban *Anolis* species, *A. carolinensis*, and the latest common ancestor of the three trunk–crown species that live in the hot‐open habitat. The alignment used to calculate the conservation and acceleration scores had a total length of 571,365,933 bases, and the alignment coverage over the reference *A. carolinensis* genome (using the established 13 chromosomes) was 52.8% (571,365,933/1,081,644,591). The alignment was divided into windows containing 10–99 bases and the conservation and acceleration scores were calculated for 9,655,872 windows. The topology of the phylogenetic tree estimated from the adjusted 4D sites (Figure 2) was consistent with previous studies (Cádiz et al., 2018; Glor et al., 2005; Losos, 2009; Nicholson et al., 2005; Poe et al., 2017). The cumulative distribution of conservation and acceleration scores across the phylogeny exhibited a higher percentage of positive CDS scores (Figure 3) indicating presence of more conservative regions in the CDS than in other annotation categories. The annotation categories exhibited minimal or no difference in the cumulative distribution of conservation and acceleration scores for each lineage (Figure S1). Extensively conserved/accelerated genomic regions were not present at any specific location in the chromosomes as shown by Manhattan plots generated for the conservation and acceleration scores (Figure S2).

### 3.2 All‐branch conserved and accelerated regions in the lineage of interest

**FIGURE 4:**
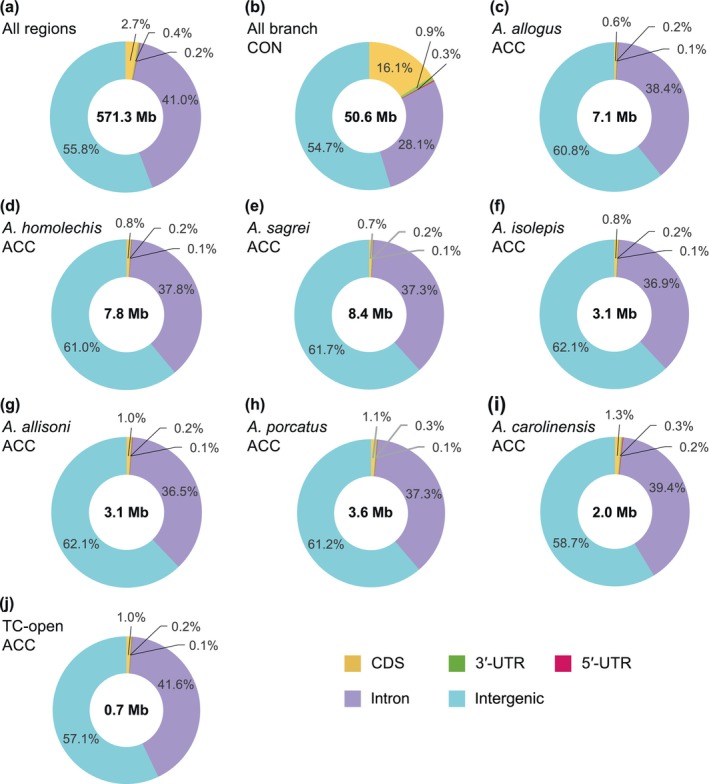
Percentage of each annotation categories in all‐branch CEs and ARs. (a) All aligned regions. (b) All‐branch CEs. (c–j), ARs in (c) *A. allogus*, (d) *A. homolechis*, (e) *A. sagrei*, (f) *A. isolepis*, (g) *A. allisoni*, (h) *A. porcatus*, (i) *A. carolinensis*, and (j) TC‐open. Color legends: CDS = yellow, 3′‐UTR = green, 5′‐UTR = magenta, intron = violet, and intergenic = cyan. 3′‐UTR, 3′‐untranslated region; 5′‐UTR, 5′‐untranslated region; AR, accelerated region; CDS, protein coding sequence; CE, conserved element; TC, trunk–crown clade.

Following FDR correction for the conservation and acceleration *p*‐values, all‐branch CEs as regions with a high average degree of conservation across all branches of phylogeny and ARs with a high nucleotide substitution rate in the branch of interest (each Cuban species, *A. carolinensis*, and the common ancestor of three trunk–crown species that occurred in the hot‐open habitat) were detected using an FDR <0.05. The total length of all‐branch CEs was 50,570,632 bases, which was 8.85% (50,570,632/571,258,887) of the total alignment. The percentage of CDS in all‐branch CEs (Figure 4b) was greater than in all aligned regions (Figure 4a). The percentage of noncoding regions (the sum of 3′‐UTR, 5′‐UTR, intron, and intergenic) in all‐branch CEs was >80%. Significantly higher proportion of intergenic sequences within noncoding regions was seen in all‐branch CEs than in all aligned regions (*χ* ^2^ test, *χ* ^2^ = 1,145,557, df = 1, and *p* < 2.2 × 10^−16^).

The CDS percentage was lower and the percentage of intergenic regions was higher in ARs than in all aligned regions and all‐branch CEs (Figure 4). The total length of ARs and the number of genes containing or flanking ARs for trunk–ground species lineages were higher than those for trunk–crown species lineages (Table 1a). In any lineage, 5% of ARs overlapped with all‐branch CEs (Table 1b). Significantly greater proportion of ncRNA genes was contained in ARs than all aligned regions (Fisher's exact test, *p* < .05, Table 2).

**TABLE 1:** The number of genes that contain or are near ARs (a) and the number of genes that contain or are near ARs that overlap with all‐branch CEs (b).

	Total length (base)	Number of genes					
		CDS	3′‐UTR	5′‐UTR	Intron	Intergenic	
						~Upstream 5 kb	~Downstream 5 kb
**(a)**							
*A. allogus*	7,133,923	407	173	106	6274	2490	2831
*A. homolechis*	7,800,289	516	172	103	6335	2670	2877
*A. sagrei*	8,426,253	482	162	93	6441	2696	2870
*A. isolepis*	3,065,048	221	73	37	4416	1175	1258
*A. allisoni*	3,126,763	248	96	50	4272	1189	1247
*A. porcatus*	3,626,579	300	97	51	4187	1305	1374
*A. carolinensis*	1,958,054	276	83	54	3621	872	1037
TC‐Open	736,220	90	26	12	2600	382	472
**(b)**							
*A. allogus*	32,546	71	4	2	166	7	33
*A. homolechis*	28,132	102	5	0	114	9	30
*A. sagrei*	25,475	67	2	2	131	5	21
*A. isolepis*	28,334	37	4	1	134	10	24
*A. allisoni*	33,035	51	7	0	147	10	32
*A. porcatus*	20,183	34	0	1	78	10	22
*A. carolinensis*	40,519	66	5	1	185	18	37
TC‐open	4827	8	1	0	30	1	2

Abbreviations: AR, accelerated region; CE, conserved element.

**TABLE 2:** Number and proportion of all‐branch CEs or ARs containing ncRNA.

	Number of regions	Number of regions overlapping ncRNA genes	Proportion of regions containing ncRNA (%)	*p*‐value
All aligned regions	9,655,872	269,314	2.79	‐
All‐branch CEs	842,688	23,644	2.81	.331
ARs				
Trunk–Ground				
*A. allogus*	121,461	4134	3.40	8.61E‐37
*A. homolechis*	132,887	4710	3.54	6.82E‐59
*A. sagrei*	143,540	4980	3.47	2.65E‐52
Trunk–Crown				
*A. isolepis*	52,357	2005	3.83	5.32E‐43
*A. allisoni*	53,312	1981	3.72	2.12E‐35
*A. porcatus*	61,666	2235	3.62	1.06E‐33
*A. carolinensis*	32,880	1021	3.11	5.83E‐04
TC‐open	12,395	398	3.21	4.93E‐03

*Note*: All‐branch CEs or ARs with >80% of the sequences that overlapped with ncRNA (snRNA, lncRNA, miRNA, rRNA, snoRNA, and scRNA) were counted. *p*‐values for Fisher's exact probability test for all aligned regions versus all‐branch CEs or ARs are shown.

Abbreviations: AR, accelerated region; CE, conserved element; lncRNA, long noncoding RNA; miRNA, microRNA; ncRNA, noncoding RNA; rRNA, ribosomal RNA; scRNA, small cytoplasmic RNA; snoRNA, small nucleolar RNA; snRNA, small nuclear RNA.

### 3.3 Genes containing or near accelerated regions

Table S2 lists all the genes containing or near (≤5 kb) ARs in each *Anolis* lineage by annotation category. For subsequent intron and intergenic analyses, we considered all‐branch CEs to represent putative functional elements and used ARs that overlapped with all‐branch CEs listed in Table 1b. Table S3 shows the GO analysis. *A. allogus*, *A. homolechis*, *A. sagrei*, *A. isolepis*, *A. allisoni*, *A. porcatus*, and *A. carolinensis* were included in the results with enrichment of at least one GO term. GO terms commonly detected in two or more lineages are shown in Table 3. GO terms detected between species belonging to different clades but having similar habitats included “cell–cell adhesion via plasma‐membrane adhesion molecules (GO:0098742)” and “homophilic cell adhesion via plasma‐membrane adhesion molecules (GO:0007156),” which were common among *A. sagrei*, *A. allisoni*, *A. porcatus*, and *A. carolinensis* in the hot‐open lineages.

**TABLE 3:** Enrichment of GO terms commonly detected in multiple lineages.

GO biological process complete	Species	Number of species
Anatomical structure development (GO:0048856)	*A. allogus*, *A. homolechis*, *A. sagrei*, *A. isolepis*, *A. allisoni*, and *A. carolinensis*	6
Anatomical structure morphogenesis (GO:0009653)	*A. allogus*, *A. homolechis*, *A. sagrei*, *A. isolepis*, and *A. allisoni*	5
Animal organ development (GO:0048513)	*A. allogus*, *A. homolechis*, *A. sagrei*, *A. allisoni*, and *A. carolinensis*	5
Animal organ morphogenesis (GO:0009887)	*A. allogus* and *A. sagrei*	2
Biological regulation (GO:0065007)	*A. allogus*, *A. homolechis*, *A. sagrei*, *A. isolepis*, *A. allisoni*, *A. porcatus*, and *A. carolinensis*	7
Biological_process (GO:0008150)	*A. allogus*, *A. homolechis*, *A. sagrei*, *A. isolepis*, *A. allisoni*, *A. porcatus*, and *A. carolinensis*	7
Cell adhesion (GO:0007155)	*A. sagrei*, *A. isolepis*, *A. allisoni*, *A. porcatus*, and *A. carolinensis*	5
Cell communication (GO:0007154)	*A. homolechis*, *A. sagrei*, and *A. allisoni*	3
Cell development (GO:0048468)	*A. homolechis*, *A. sagrei*, and *A. isolepis*	3
Cell differentiation (GO:0030154)	*A. homolechis*, *A. sagrei*, *A. isolepis*, and *A. carolinensis*	4
Cell junction organization (GO:0034330)	*A. isolepis* and *A. allisoni*	2
Cell morphogenesis (GO:0000902)	*A. sagrei* and *A. isolepis*	2
Cell morphogenesis involved in differentiation (GO:0000904)	*A. sagrei* and *A. isolepis*	2
Cell morphogenesis involved in neuron differentiation (GO:0048667)	*A. sagrei* and *A. isolepis*	2
Cell part morphogenesis (GO:0032990)	*A. sagrei* and *A. isolepis*	2
Cell projection morphogenesis (GO:0048858)	*A. sagrei* and *A. isolepis*	2
Cell–cell adhesion (GO:0098609)	*A. isolepis*, *A. allisoni*, *A. porcatus*, and *A. carolinensis*	4
Cell–cell adhesion via plasma‐membrane adhesion molecules (GO:0098742)	*A. sagrei*, *A. allisoni*, *A. porcatus*, and *A. carolinensis*	4
Cellular component organization (GO:0016043)	*A. allogus*, *A. homolechis*, *A. sagrei*, and *A. carolinensis*	4
Cellular component organization or biogenesis (GO:0071840)	*A. allogus*, *A. homolechis*, *A. sagrei*, and *A. carolinensis*	4
Cellular developmental process (GO:0048869)	*A. homolechis*, *A. sagrei*, *A. isolepis*, and *A. carolinensis*	4
Cellular metabolic process (GO:0044237)	*A. homolechis* and *A. allisoni*	2
Cellular process (GO:0009987)	*A. allogus*, *A. homolechis*, *A. sagrei*, *A. isolepis*, *A. allisoni*, *A. porcatus*, and *A. carolinensis*	7
Cellular response to stimulus (GO:0051716)	*A. homolechis*, *A. sagrei*, *A. allisoni*, and *A. porcatus*	4
Developmental process (GO:0032502)	*A. allogus*, *A. homolechis*, *A. sagrei*, *A. isolepis*, *A. allisoni*, and *A. carolinensis*	6
Homophilic cell adhesion via plasma membrane adhesion molecules (GO:0007156)	*A. sagrei*, *A. allisoni*, *A. porcatus*, and *A. carolinensis*	4
Intracellular signal transduction (GO:0035556)	*A. homolechis* and *A. allisoni*	2
Macromolecule metabolic process (GO:0043170)	*A. homolechis* and *A. sagrei*	2
Macromolecule modification (GO:0043412)	*A. homolechis* and *A. sagrei*	2
Metabolic process (GO:0008152)	*A. allogus*, *A. homolechis*, *A. sagrei*, and *A. isolepis*	4
Multicellular organism development (GO:0007275)	*A. allogus*, *A. homolechis*, *A. sagrei*, *A. isolepis*, *A. allisoni*, and *A. carolinensis*	6
Multicellular organismal process (GO:0032501)	*A. allogus*, *A. homolechis*, *A. sagrei*, *A. isolepis*, *A. allisoni*, and *A. carolinensis*	6
Nervous system development (GO:0007399)	*A. isolepis*, *A. allisoni*, and *A. carolinensis*	3
Neuron development (GO:0048666)	*A. sagrei* and *A. isolepis*	2
Neuron projection development (GO:0031175)	*A. sagrei* and *A. isolepis*	2
Neuron projection morphogenesis (GO:0048812)	*A. sagrei* and *A. isolepis*	2
Nitrogen compound metabolic process (GO:0006807)	*A. homolechis* and *A. sagrei*	2
Organic substance metabolic process (GO:0071704)	*A. allogus*, *A. homolechis*, *A. sagrei*, and *A. isolepis*	4
Organonitrogen compound metabolic process (GO:1901564)	*A. homolechis*, *A. sagrei*, and *A. isolepis*	3
Plasma membrane bounded cell projection morphogenesis (GO:0120039)	*A. sagrei* and *A. isolepis*	2
Primary metabolic process (GO:0044238)	*A. homolechis*, *A. sagrei*, and *A. isolepis*	3
Protein metabolic process (GO:0019538)	*A. homolechis*, *A. sagrei*, and *A. isolepis*	3
Protein modification process (GO:0036211)	*A. homolechis* and *A. sagrei*	2
Regulation of biological process (GO:0050789)	*A. allogus*, *A. homolechis*, *A. sagrei*, *A. isolepis*, and *A. allisoni*	5
Regulation of biological quality (GO:0065008)	*A. homolechis* and *A. sagrei*	2
Regulation of cellular process (GO:0050794)	*A. allogus*, *A. homolechis*, *A. sagrei*, and *A. allisoni*	4
Regulation of developmental process (GO:0050793)	*A. allogus* and *A. homolechis*	2
Regulation of response to stimulus (GO:0048583)	*A. homolechis* and *A. sagrei*	2
Response to stimulus (GO:0050896)	*A. homolechis*, *A. sagrei*, *A. allisoni*, *A. porcatus*	4
Signal transduction (GO:0007165)	*A. homolechis* and *A. allisoni*	2
Signaling (GO:0023052)	*A. homolechis*, *A. sagrei*, and *A. allisoni*	3
Synapse organization (GO:0050808)	*A. isolepis* and *A. allisoni*	2
System development (GO:0048731)	*A. allogus*, *A. homolechis*, *A. sagrei*, *A. isolepis*, *A. allisoni*, *A. porcatus*, and *A. carolinensis*	7

*Note*: Of the GO terms shown in Table S3, those detected in two or more lineages are shown. GO, Gene ontology.

Abbreviation: GO, Gene ontology.

### 3.4 Genes commonly accelerated in lineages with similar thermal habitats

To search for genes that are important for adaptation to thermal habitats, we extracted the common genes containing ARs for lineages living in hot‐open or cool‐shaded habitats: A common ancestral branch of three hot‐open species belonging to the trunk–crown clade (TC‐open) and *A. sagrei* from the trunk–ground clade were used as two hot‐open lineages, and *A. isolepis* and *A. allogus* were used as two cool‐shaded lineages. A total of 41 and 96 genes were identified in the hot‐open and cool‐shaded habitat lineages, respectively, where acceleration was commonly detected in either the CDS, 3′‐UTR, 5′‐UTR, intron, or regions within the upstream or downstream 5 kb (Table S4); no significant GO term enrichment was identified in these genes. Table S5 summarizes gene functions commonly accelerated in lineages with similar habitats. Common genes in the hot‐open lineages included a gene (*BRCA2*) associated with DNA repair. Genes related to circadian rhythm regulation, sleep, and lipid metabolism were detected for the hot‐open and cool‐shaded habitat lineages. In addition, several genes are involved in eye tissue formation, and several genes such as *GRIK4*, *PIK3C3*, *POU6F2*, *SYNJ1*, *IMPG1*, E*XT2*, *CTBP2*, *FGF19*, and *CDH4* are expressed in the retina and may be implicated in photoreception or visual formation. In addition, genes that were expressed in the central nervous system and involved in behavioral phenotypes were identified in both hot‐open and cool‐shaded lineages. For several genes, the same windows in the multiple alignments were accelerated among different lineages with similar thermal habitats, including two and six genes in the hot‐open and cool‐shaded lineages, respectively (Table S4, genes marked with “✓”). Of the genes detected in the hot‐open lineages, *SNRNP35*, which encodes a subunit of the spliceosome complex, is involved in U12 splicing and fluctuates in response to stress hormones in the mouse brain (Huckins et al., 2020). In the cool‐shaded lineages, *COL5A1* encodes V‐type collagen and is important in the formation of skin (Wenstrup et al., 2006) and cornea (Sun et al., 2011), *PIK3CD* encodes a lipid kinase that functions in the PI3K pathway and is involved in the immune response (Tangye et al., 2019), and *HIP1R* is expressed in various brain regions and is involved in excitatory synapse formation (Peng et al., 2017), were present.

## 4 DISCUSSION

This study investigated evolutionarily conserved and accelerated genomic regions using whole genomes of eight *Anolis* lizard species that have repeatedly adapted to similar thermal environments in multiple lineages, providing previously unknown candidate regions for thermal adaptation. The proportion of CDS in all‐branch CEs was larger than that in the aligned regions. The cumulative distribution of conserved and accelerated scores over all lineages indicated toward existence of more conserved regions in CDS. Similar trends of CEs in various animal species were reported previously, reflecting on the functional importance of CDS in the genome (Siepel et al., 2005). The proportion of noncoding regions in all‐branch CEs was still >80%. The intergenic proportion within noncoding regions was significantly larger in all‐branch CEs than in all aligned regions. The length of the intergenic region associates with the complexity of gene expression, and these regions contain several sequences essential for gene expression regulation (Nelson et al., 2004). Conserved noncoding regions found in other studies were expected to include regulatory sequences, such as promoters, enhancers, and CTCF‐binding regions involved in three‐dimensional structural genome formation (Christmas et al., 2023; Hemberg et al., 2012; Pennacchio et al., 2006; Snetkova et al., 2022; Woolfe et al., 2005). In this analysis, all regions other than those annotated as “gene” were classified as intergenic; therefore, the conserved intergenic regions found in this study may include various previously unknown functional regions as described above. The actual function of each region can be elucidated in future research including information on different spatiotemporal chromatin accessibility. The conserved regions identified in this study could be a potential source for identifying novel functional regions. Christmas et al. (2023) categorized conserved bases among mammals into various functional annotations and reported low enrichment of bases present in introns. Similarly, the relative lack of intron conservation may have increased the relative proportion of intergenic regions in our results.

Fast‐evolving genomic regions of each lineage, such as ARs, had a lower proportion of CDS and a higher proportion of intergenic regions than all‐branch CEs, which has been confirmed in other studies (Kostka et al., 2018; Pollard et al., 2006). Gene regulatory regions evolve more rapidly than coding regions (Kaplow et al., 2022; Villar et al., 2015; Wong et al., 2020) because they are flexible and can maintain similar functions in gene regulation without strict sequence conservation. During adaptive evolution, accelerated sequence alterations in gene regulatory regions may have contributed to the fine‐tuning of spatiotemporal expression patterns and levels without altering the gene structure. The proportion of ARs containing ncRNAs was significantly higher in any lineage compared with the entire aligned region. Pang et al. (2006) reported lack of significant sequence conservation in certain ncRNAs, which is consistent with our results. The lncRNA sequences evolve more rapidly than protein‐coding genes (Mattick et al., 2023) and species‐specific transcription of intergenic lncRNAs has been correlated with the upregulation of near protein‐coding genes in rodents (Kutter et al., 2012). Overall, these findings indicate several unknown functional elements in the noncoding ARs affected by positive natural selection or relaxation of negative selection (Hubisz & Pollard, 2014). In each lineage, genes containing or near ARs exhibited functional bias in several annotation categories, which may play crucial role in affecting the unique phenotype of each lineage. Several GO terms were commonly found in multiple species lineages suggested the essential role of these processes in *Anolis* species evolution. Notably, this alignment is incomplete and captures only a portion of the accelerated evolution across the genome. Only 52.8% of the entire reference *A. carolinensis* genome by multiple alignments was covered while the remaining sequence information was lost in this analysis. The homologous regions among the species were extracted while performing the alignment. Therefore, several regions that failed to align may contain rapidly evolving elements.

Herein, we identified completely novel candidate genes that function in thermal adaptation and that differ from genes where nonsynonymous mutations were estimated to be under positive natural selection in the CDS in hot‐open lineages of Cuban *Anolis* lizards (Kanamori et al., 2021). Kanamori et al. (2021) searched genes under positive selection for each nonsynonymous site based on the ratio of synonymous to nonsynonymous substitution rates. This method can detect single amino acid mutations that significantly affect adaptation. Conversely, in this study, the acceleration trend across the nucleotide sequence was calculated for each window separated by 10–99 bases. This can detect natural selection beyond a single site and include information on the regional context, which varies in scope from Kanamori et al. (2021). For example, this analysis can target natural selection for synonymous sites in the CDS that can affect mRNA levels and translation, splicing regulatory elements, and ncRNA target sites (Lin et al., 2011; Shen et al., 2022). This analysis conducted from a novel perspective could uncover previously overlooked positively selected genes. We consolidated the number of genes thought to have contributed to adaptation to the thermal habitat by detecting those that commonly contain ARs among lineages that live in similar environments. Cases where acceleration traces were observed in the same window in two lineages as well as those where ARs existed in different locations within or near the same gene were considered. The window width used for this analysis was 10–99 bases to ensure sufficient detection power and avoid obscuring the acceleration and conservation signals (see Section 2). Therefore, the scope of our analysis was limited to the window size and the common gene detection method. Convergent evolution across genes can also be examined by other techniques, such as the RERconverge package (Kowalczyk et al., 2019), although these were not included herein because of their different scope.

Genes that were commonly accelerated in two lineages inhabiting similar thermal habitats (i.e., two lineages inhabiting the hot‐open or cool‐shaded habitats) were detected. These genes included one related to DNA repair in hot‐open lineages and several involved in circadian rhythms, sleep, vision, lipid metabolism, and behavior in hot‐open and cool‐shaded lineages. Species inhabiting hot‐open environments may be exposed to more intense sunlight, and *BRCA2* may function to compensate for UV‐induced DNA damage (Rastogi et al., 2010). Temperature, light environment, and circadian rhythm are closely related. The circadian system maintains synchrony between the internal cycle of the organism and cycles present in the environment, such as light and temperature, and regulates daily fluctuations in behavior and physiological functions (Paul et al., 2009; Seebacher & Franklin, 2005). In Drosophila, thermocycle and photocycle entrainments generate similar circadian expression profiles in the head, and light and temperature coordinately entrain molecular and behavioral circadian rhythms (Boothroyd et al., 2007). In this analysis, we detected multiple genes that are expressed in the retina and are expected to be involved in photoreception. The retina transmits light information to the brain and has an independent circadian oscillator (Tosini et al., 2001). Thermoregulatory behaviors, such as basking, are considered to follow daily cycles related to temperature and light (Díaz, 1991; Huey & Pianka, 1977; Scheers & Van Damme, 2002), and alterations in light‐sensing and circadian regulation function may be associated with the formation of various daily thermoregulatory behavioral patterns synchronized with the photoperiod. Among the species used in this study, those that inhabit hot‐open environments are heliothermic and obtain thermal energy from sunlight, whereas those that inhabit cool‐shaded environments are nonheliothermic. The tendency for species living in cooler forest environments to bask less often than species living in open environments has also been observed in anoles on other islands (Huey & Webster, 1976). Therefore, the close relationship between the light environment of the habitat and regulation of body temperature using sunlight is common to some extent, at least in the genus *Anolis*. *Anolis cristatellus*, an open species, exhibits thermoregulation behavior based on light intensity, whereas *A. gundlachi*, a forest species, exhibits behavioral patterns independent of light intensity (Hertz et al., 1994). Moore and Menaker (2012) found that circadian responses to light significantly differed in four *Anolis* lizard species that are phylogenetically related but experience different body temperatures, each with varying amounts of sunlight in their habitat. Akashi et al. (2016) also suggested that genes involved in circadian rhythms play an essential role in thermal adaptation in trunk–ground *Anolis* lizards in Cuba. Numerous studies have shown that environmental temperature affects lipid metabolism in ectotherms (Pafilis et al., 2007; Plasman et al., 2019; Shen et al., 2005). A meta‐analysis integrating several studies investigating genes involved in thermal adaptation confirmed that the GO terms of energy metabolism and lipid metabolism were well detected in several studies (Porcelli et al., 2015). In the desert iguana (*Dipsosaurus dorsalis*), differences in dietary fatty acid composition alter preference temperature (Simandle et al., 2001). Genes related to lipid metabolism were also implicated in thermal adaptation in *Anolis* lizards in a comparison of genomes across populations at varying elevations (Rodríguez et al., 2017). Endothermic species adjust metabolic heat production in response to alterations in environmental heat load, and metabolic adaptation to the thermal environment in ectotherms appears to partly depend on the same regulatory pathways as adaptive heat production in endotherms (Seebacher, 2009). In addition, lipid metabolism may be closely related to the circadian rhythm. The rhythm of food intake and energy metabolism is diurnally regulated (Gooley, 2016), and circadian rhythm disruption is associated with eating behavior, altered metabolic function, and weight gain in mammals (Touitou et al., 2017). Artificial light perturbation of the photo‐rhythm at night increases fat accumulation in *A. carolinensis* (Taylor et al., 2022). In addition, in humans, diet nutrient composition, particularly fatty acids and glucose, as well as the timing of meals, may influence the circadian clock (Oosterman et al., 2015). Chang et al. (2021) also reported that temperature changes affect circadian rhythms and lipid metabolic pathways in the Mongolia racerunner (*Eremias argus*). Our results indicate that alterations in light‐sensing function, circadian rhythm regulation, and lipid metabolism directly or indirectly influenced each other and contributed to the adaptation of *Anolis* lizards to environments containing different temperatures and sunlight levels.

Behavior is the primary means of thermoregulation in ectotherms that depend on external heat sources for body temperature (Bodensteiner et al., 2021). The cost of thermoregulatory behavior, such as energy consumption for movement and evading predator encounters, differs between hot‐open habitats with easy access to heat sources and cool‐shaded habitats with almost no direct sunlight (Losos, 2009). *A. allisoni*, *A. porcatus*, *A. sagrei*, and *A. homolechis* are thermoregulatory active, whereas *A. allogus* is a thermoconformer (Schettino, 1999), and their strategies for thermoregulatory behavior likely differ depending on the environment. In *A. carolinensis*, many genes involved in nervous system development and behavior underwent positive selection in populations that recently expanded from Florida to the northern temperate zone (Bourgeois & Boissinot, 2019). Although the adaptive functions of the behavioral genes identified in our study are unknown, changes in these genes may contribute to behavioral adaptation to the environment of Cuban *Anolis* lizards. In this analysis, no enrichment in the function of genes common to lineages with similar habitats was detected. Only a small number of genes were commonly detected at this time, and significant enrichment would only be found if the functions were significantly biased. Alternatively, as mentioned above, the gene functions involved in thermal adaptation are wide‐ranging, including UV protection, circadian rhythm, lipid metabolism, and behavior. Therefore, enrichment of GO terms may not occur with this number of genes.

Several genes, including *PIK3C3*, *CA13*, *SNRNP35*, *MX1*, *QRICH2*, *NINJ2*, *THSD7B*, *MTR*, *PACRG*, *PTK7*, *OBSCN*, *MUC6*, *ABCA12*, and *DNAH11*, associated with the AR in our study were also detected as DEGs at different temperatures in three Cuban *Anolis* lizards (*A. allogus*, *A. homolechis*, and *A. sagrei*) inhabiting different thermal habitats (Akashi et al., 2016). These common DEGs whose expression changed according to the difference in temperature may play a particularly important role in thermal adaptation and may have evolved through altered gene expression patterns to adapt to different thermal environments.

The neutral evolutionary model was estimated using modified 4D sites; however, the sites to be designated as neutral remains unclear. Although 4D sites and ancestral repeats are widely used to estimate neutral substitution rates (Pheasant & Mattick, 2007), differences in substitution rates between these regions have been reported (Künstner et al., 2011). Alignment of ancestral repeats is more difficult as branches become deeper, which reduces the accuracy of the neutral model (Pheasant & Mattick, 2007). Some ancestral repeats may be targeted for selection, and their replacement rate is not constant (Pheasant & Mattick, 2007). Synonymous sites have been recently suggested to be subjected to selection (Hanson & Coller, 2018), and all 4D sites may not be neutral. Herein, we found biased use of 4D sites in the *Anolis* lizard genome; therefore, we excluded codon sets during neutral model building that had an extreme frequency of use bias. However, further discussion is needed regarding a more accurate methodology for estimating neutral models, and the development of novel methods is required in future.

This study focused on species that are believed to have converged or parallelly evolved into similar thermal environments among the genus *Anolis*, belonging to two different ecomorphs inhabiting in Cuba. This could help in detecting genetic regions associated with adaptation to the thermal environment, independent of the ecomorphs evolution or other characteristic traits of each species. Comparison within the same genus minimized the differences in genomes caused by differences in other traits. However, involvement of same genes in the thermal adaptation of species other than *Anolis* remains unclear.

Other genes important for thermal adaptation would have remained missing in this analysis. Corbett‐Detig et al. (2020) reported that phenotypic convergence was not necessarily driven by convergence at the protein sequence level in *Anolis* lizards. Similarly, different genes in each lineage may contribute to adaptation to similar thermal environments, and those required for thermal adaptation may independently accelerate in each lineage. In addition, the ancestral habitat of each lineage remains unknown; thus, adaptive changes to the environment may have occurred before the branch in which acceleration was detected.

These analyses were sequence data‐based with no direct evidence indicating that the identified regions are associated with adaptation to the thermal niche. Several possible scenarios exist where the genes detected in this study do not contribute to thermal adaptation. Some regions undergoing accelerated evolution may be related to adaptation to factors such as sexual selection, interspecific relationships, or conflicts between genes, rather than the ecological environment. For example, same gene may coincidentally function in adaptations to different aspects of each lineage and may be detected as a common gene. Furthermore, genes contributing to adaptation to similar thermal environments habitats may not necessarily have only temperature‐related functions. Factors such as humidity, light, and a type of food are expected to differ between habitats; however, as mentioned above, adaptation to these factors are closely related. Although it was difficult to separate these differences completely in this study, candidates believed to be related to the temperature environment based on gene function were selected. This study identified candidate genes relevant to thermal microhabitat adaptation for future studies. Confirming the function of each gene in temperature response using direct methods such as genome‐editing technology is warranted in future studies. The importance of this analysis lies in comprehensive consolidation of the candidate regions believed to have contributed to thermal adaptation from the entire genome, including noncoding regions. Multiple common genes from different lineages were identified, and the functions of several of these genes were considered to play a crucial role in adaptation to similar thermal environments.

## 5 CONCLUSION

Herein, we identified genomic regions predicted to be associated in thermal adaptive evolution from the whole genomes of eight *Anolis* lizard species. The common genes containing or near ARs between lineages with similar habitats were detected. Several of these genes were related to circadian rhythm and behavior, which is consistent with previous studies suggesting the importance of circadian rhythm and behavioral thermoregulation in thermal adaptation. The functions of the candidate genomic regions involved in thermal adaptation remains unknown and in vivo evaluation is warranted. This study provides a foundation for comprehensive elucidation of the genetic basis of thermal adaptation across the entire *Anolis* genome.

## AUTHOR CONTRIBUTIONS

**Fuku Sakamoto:** Conceptualization (supporting); data curation (lead); formal analysis (lead); investigation (equal); software (equal); visualization (lead); writing – original draft (lead); writing – review and editing (lead). **Shunsuke Kanamori:** Data curation (supporting); formal analysis (supporting); methodology (supporting); software (supporting); writing – review and editing (supporting). **Luis M. Díaz:** Investigation (supporting); resources (supporting); writing – review and editing (supporting). **Antonio Cádiz:** Investigation (supporting); resources (supporting); writing – review and editing (supporting). **Yuu Ishii:** Data curation (supporting); formal analysis (supporting); writing – review and editing (supporting). **Katsushi Yamaguchi:** Data curation (supporting); resources (supporting); writing – review and editing (supporting). **Shuji Shigenobu:** Data curation (supporting); resources (supporting); writing – review and editing (supporting). **Takuro Nakayama:** Data curation (supporting); formal analysis (supporting); software (equal); writing – review and editing (supporting). **Takashi Makino:** Data curation (supporting); formal analysis (supporting). **Masakado Kawata:** Conceptualization (lead); data curation (supporting); funding acquisition (lead); project administration (lead); resources (supporting); writing – original draft (lead); writing – review and editing (lead).

## CONFLICT OF INTEREST STATEMENT

The authors declare no conflicts of interest.

## Supporting information


Figures S1–S2

Tables S1–S5

## Data Availability

Pairwise sequence alignment, multiple sequence alignment, neutral phylogenetic tree, conservation/acceleration score data, and python scripts generated in this study are available at Dryad (https://doi.org/10.5061/dryad.31zcrjdrk).
