# Supplementary material for: Detection of evolutionary conserved and accelerated genomic regions related to adaptation to thermal niches in Anolis lizards
Source: Ecol Evol. 2024 Mar 7;14(3):e11117. doi: 10.1002/ece3.11117 (PMC10920033; doi:10.1002/ece3.11117)
Supplement: Supplementary file 1 — Figures S1–S2 [file ECE3-14-e11117-s001.pdf]

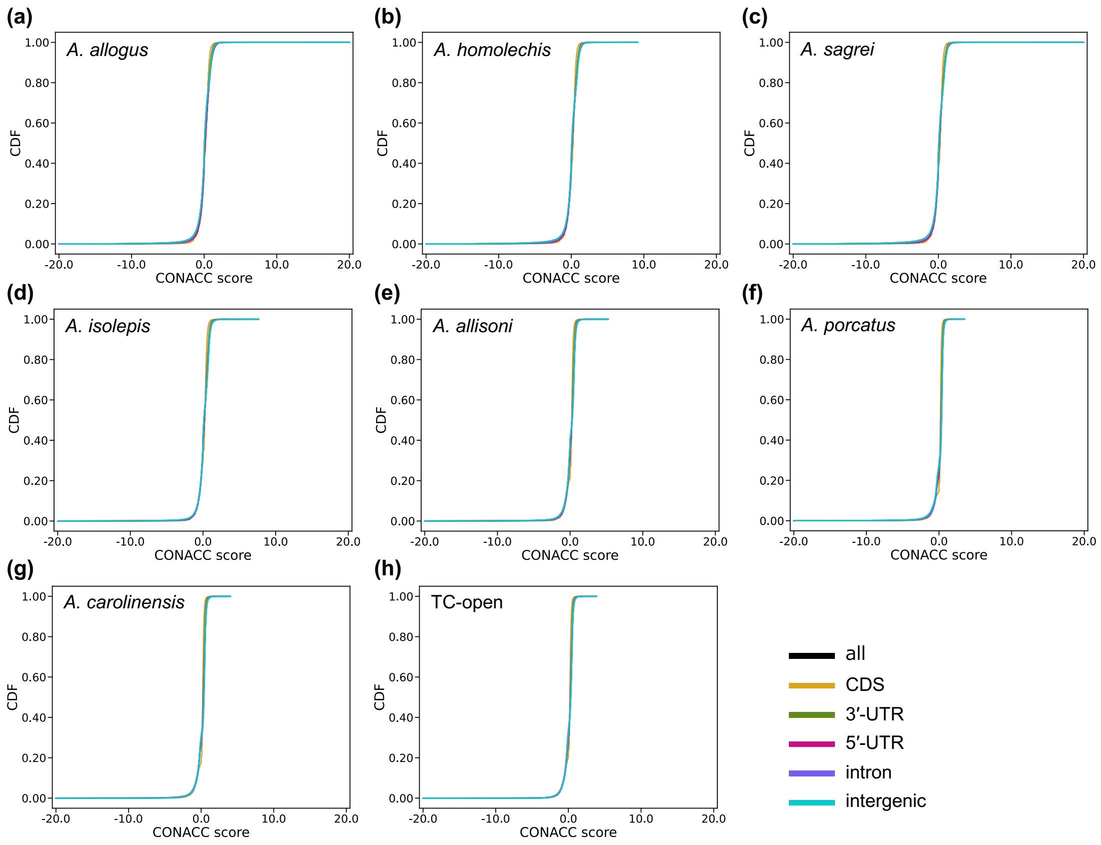


## FIGURE S1

Cumulative distribution frequency (CDF) of CONACC scores (conservation-acceleration scores) by annotation category for each lineage. CONACC scores of (a) *A. allogus*, (b) *A. homolechis*, (c) *A. sagrei*, (d) *A. isolepis*, (e) *A. allisoni*, (f) *A. porcatus*, (g) *A. carolinensis*, and (h) TC-open. Positive larger CONACC scores represent more conserved and negative larger CONACC scores represent more acceleration. Color legends: all regions = black, CDS = yellow, 3′-UTR = green, 5′-UTR = magenta, intron = violet, and intergenic = cyan. CDS, protein coding sequence; 3′-UTR, 3′-untranslated region; 5′-UTR, 5′-untranslated region.

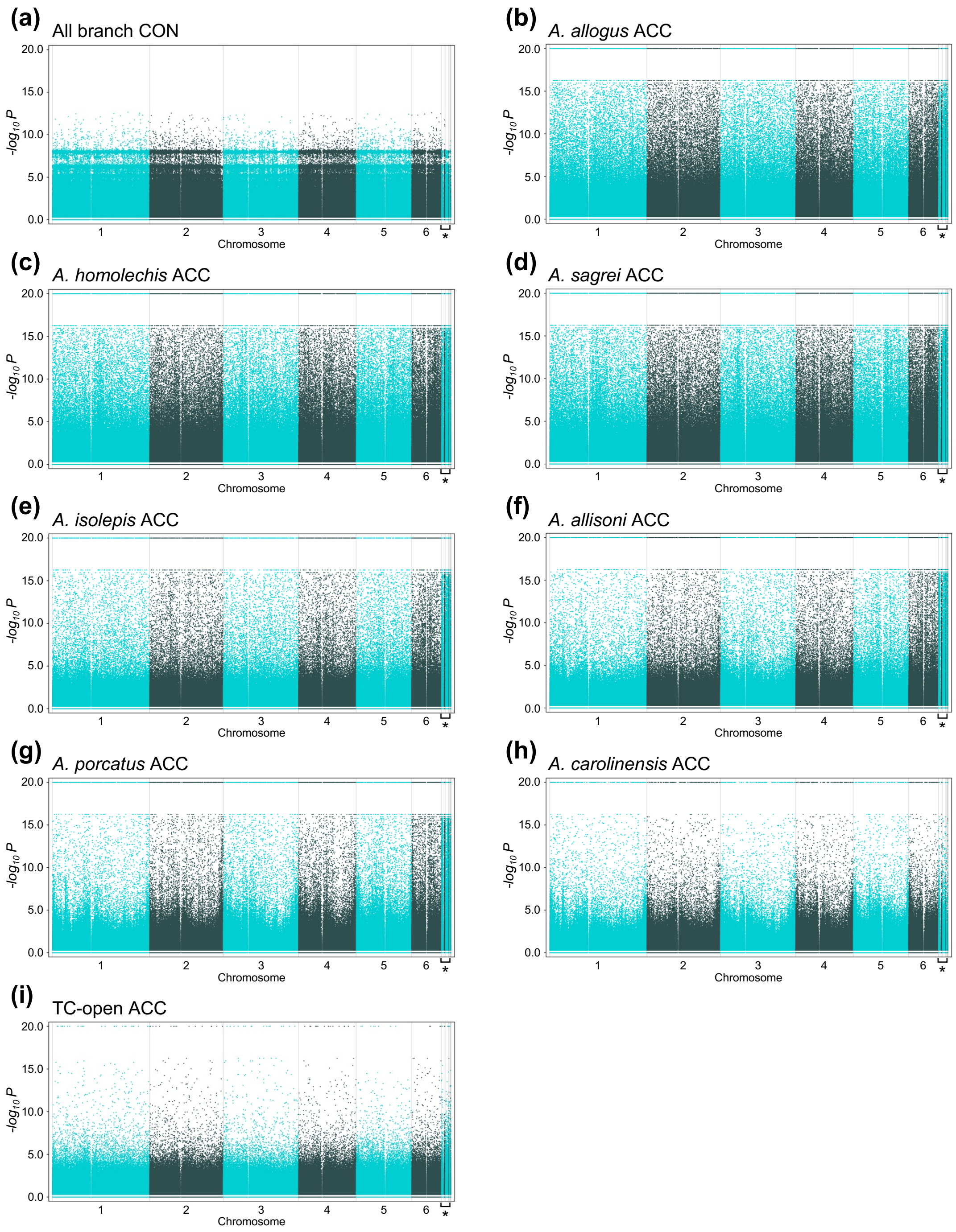


## FIGURE S2

Manhattan plot for conservation and acceleration scores on chromosomes. (a) Conservation scores for all branches. (b–i) Acceleration scores for (b) *A. allogus*, (c) *A. homolechis*, (d) *A. sagrei*, (e) *A. isolepis*, (f) *A. allisoni,* (g) *A. porcatus*, (h) *A. carolinensis*, and (i) TC-open. "*" includes scaffold a, b, c, d, f, g, and h. The $-\log_{10}p$ value of the acceleration score (b–i) appears

to have reached the computational upper limit value of 20. In a range closer to $-\log_{10}p = 20$, the score might not be calculated accurately because of the detection resolution of the software.
